# The impact of lactate clearance on outcomes according to infection sites in patients with sepsis: a retrospective observational study

**DOI:** 10.1038/s41598-021-01856-5

**Published:** 2021-11-17

**Authors:** Momoko Sugimoto, Wataru Takayama, Kiyoshi Murata, Yasuhiro Otomo

**Affiliations:** 1 Department of Emergency and Disaster Medicine, Trauma and Acute Critical Care Center, Tokyo Medical and Dental University Hospital of Medicine, 1-5-45, Yushima, Bunkyo-ku, Tokyo, 113-0034 Japan; 2 The Shock Trauma and Emergency Medical Center, Matsudo City General Hospital, 933-1 Sendabori, Matsudo, Chiba Japan

**Keywords:** Medical research, Bacterial infection

## Abstract

Whether lactate clearance (LC) influences outcomes differently depending on the infection site in sepsis cases is not fully elucidated. Herein, we analyzed LC’s clinical utility as a predictor of patient outcomes according to infection site. This retrospective study, conducted at two tertiary emergency critical care medical centers in Japan, included patients with sepsis or septic shock. The associations between infection site (lungs vs. other organs) and in-hospital mortality and ventilator-free days (VFDs) were evaluated using univariable and multivariate analyses. We assessed LC’s ability to predict in-hospital mortality using the area under the receiver operating characteristic curve. Among 369 patients with sepsis, infection sites were as follows: lungs, 186 (50.4%); urinary tract, 45 (12.2%); abdomen, 102 (27.6%); and other, 36 (9.8%). Patients were divided into a pneumonia group or non-pneumonia group depending on their infection site. The pneumonia group displayed a higher in-hospital mortality than the non-pneumonia group (24.2% vs. 15.8%, *p* = 0.051). In the multivariate analysis, lower LC was associated with higher in-hospital mortality [adjusted odds ratio (AOR), 0.97; 95% confidence interval (CI) 0.96–0.98; *p* < 0.001] and fewer VFD [adjusted difference *p* value (AD), − 1.23; 95% CI − 2.42 to − 0.09; *p* = 0.025] in the non-pneumonia group. Conversely, LC did not affect in-hospital mortality (AOR 0.99; 95% CI 0.99–1.00; *p* = 0.134) and VFD (AD − 0.08; 95% CI − 2.06 to 1.91; *p* = 0.854) in the pneumonia group. Given the differences in the impact of LC on outcomes between the pneumonia and non-pneumonia groups, this study suggests that optimal treatment strategies might improve outcomes. Further studies are warranted to validate our results and develop optimal therapeutic strategies for sepsis patients.

## Introduction

The Surviving Sepsis Campaign Guidelines 2016 have recommended guiding resuscitation to normalize lactate concentrations in patients with elevated concentrations^1^. Serum lactate concentration is widely used in the emergency department (ED) and the intensive care unit (ICU) to assess tissue perfusion^2^. Furthermore, the kinetics of lactate concentration, including both the production and elimination of lactate, may be more useful than the initial lactate concentration for assessing the outcomes of patients who present with sepsis and septic shock^3–5^. To evaluate the decrease in blood lactate concentration, lactate clearance (LC) has gained interest, and two studies have reported that impaired LC is associated with worse outcomes in patients with sepsis^6,7^. Furthermore, after adjusting for risk factors, lactate-guided therapy in ICU patients with hyperlactatemia has shown a significant association with lower hospital mortality^8^.

Fluid resuscitation has been recommended to improve LC in patients with severe sepsis^9^. However, excess fluid therapy can be harmful to the respiratory state^10^, especially in patients with severe pneumonia. Therefore, there is a need for judicious fluid therapy to achieve a more gradual improvement in LC in patients with pneumonia, thus suggesting different clinical impacts of LC between those with pneumonia and with other infections. In this study, we analyzed the performance of LC in predicting patient outcomes according to their sites of infection.

## Methods

### Study design and setting

This retrospective observational study was conducted among patients with sepsis who were admitted to two tertiary emergency critical care medical centers in Japan (Tokyo Medical and Dental University Hospital of Medicine and Matsudo City General Hospital) between April 1, 2012 and March 31, 2019.

### Ethics declarations

This study complied with the principles of the 1964 Declaration of Helsinki and its later amendments regarding reviewing and publishing information from patients’ medical records and was approved by the Institutional Review Board of Tokyo Medical and Dental University and Matsudo City Hospital (#M2020-379). The ethics committees of both participating institutes individually approved this study. The requirement for informed consent was waived because the design of the study was retrospective in nature and because of the use of anonymized patient and hospital data. The waiver was approved by the ethics committees of Tokyo Medical and Dental University and Matsudo City Hospital.

### Study population

We consecutively enrolled all adult patients who met the sepsis-3 criteria^11^ upon admission to the ED. We excluded patients who met at least one of the following criteria: (1) aged < 18 years, (2) cardiac arrest on arrival at the EDs, (3) transferred from another hospital, (4) hospitalized for less than 24 h, or (5) missing or insufficient data regarding the study variables. All included patients received treatment according to the Surviving Sepsis Campaign Guideline 2016^1^.

### Data collection

The following information was collected retrospectively from the patients’ medical records: age, sex, in-hospital mortality, and length of time that the patient was on a ventilator. In addition, we obtained laboratory results, including blood lactate concentration, upon arrival at the ED and 24 h after admission. In the two centers, these two timings of obtaining laboratory results were based on an established protocol. For all included patients, the worst Sequential Organ Failure Assessment (SOFA) score was assessed within the first 24 h of admission.

### Outcomes and definitions

Sepsis was defined as infection in addition to acute organ dysfunction, which was indicated by an increase of two or more points in the SOFA scores^11^. Infection was defined as the presence of positive cultures or clinical infection that required antibiotic administration. The site of infection was determined based on clinical, laboratory, and radiographic findings at the attending physicians’ discretion. LC (%) was defined as ([initial lactate – delayed lactate]/initial lactate) × 100, where delayed lactate was defined as the lactate concentration 24 h after the initial measurement at patient arrival. A negative value indicated an increase in lactate concentration.

The primary outcome was in-hospital mortality. The secondary outcome was ventilator-free days (VFDs)^12^.

### Statistical analysis

The included population was divided into four groups according to the site of infection: lungs, urinary tract, abdomen, and other (soft tissue, central nervous system, and undifferentiated sites of infection). We used an analysis of variance to assess the baseline characteristics of the four groups. In the univariable analysis, the continuous variables were compared using the Kruskal–Wallis test, and categorical variables were compared using Pearson’s chi-squared test. LC was classified as a continuous variable. We further divided the enrolled patients into two groups: the pneumonia group and the non-pneumonia group (patients with infections other than pneumonia). We evaluated the differences in the outcomes between the pneumonia and non-pneumonia groups, as well as assessed the differences between the survivors and non-survivors in the two groups. In the univariable analysis, the participants’ characteristics were analyzed using the Mann–Whitney U test for the continuous variables and Fisher’s exact test for categorical variables.

First, we used a multivariable logistic regression model to evaluate the interaction between LC, as a categorical variable, and pneumonia to determine whether pneumonia influenced the impact of LC on the patient outcomes. An interaction term means a variable that represents an interaction between two variables. We incorporated age, SOFA scores, and LC as variables in the multivariate model, which were selected a priori and were clinically plausible (subject-matter knowledge), and the number of outcomes (the rule of 10 events per variable). Second, to evaluate the impact of LC on the outcomes in the overall population, we used multivariate ordered logistic or linear regression analysis. Third, we further performed the analysis similarly in the pneumonia and non-pneumonia groups. Adjusted odds ratios (AOR) and adjusted differences (AD) with 95% confidence interval (CI) were calculated to assess the associations between various predictive variables and patient outcomes. AOR is an odds ratio that has been adjusted to account for other predictor variables produced by a logistic regression model, while AD is a measure of association between exposure and outcome by a linear regression analysis.

We constructed a receiver operating characteristic (ROC) curve to assess the ability of LC to predict in-hospital mortality. In addition, the area under the curve (AUC) was calculated for predictor variables in the logistic model with age, SOFA scores, and LC. We compared two different models of the AUCs (age and SOFA scores vs. age, SOFA scores, and LC). The differences were considered statistically significant at two-sided *p* values < 0.05. All statistical analyses were conducted using SPSS software, version 27 (IBM Corp., Armonk, NY, USA).

## Results

**Figure 1:**
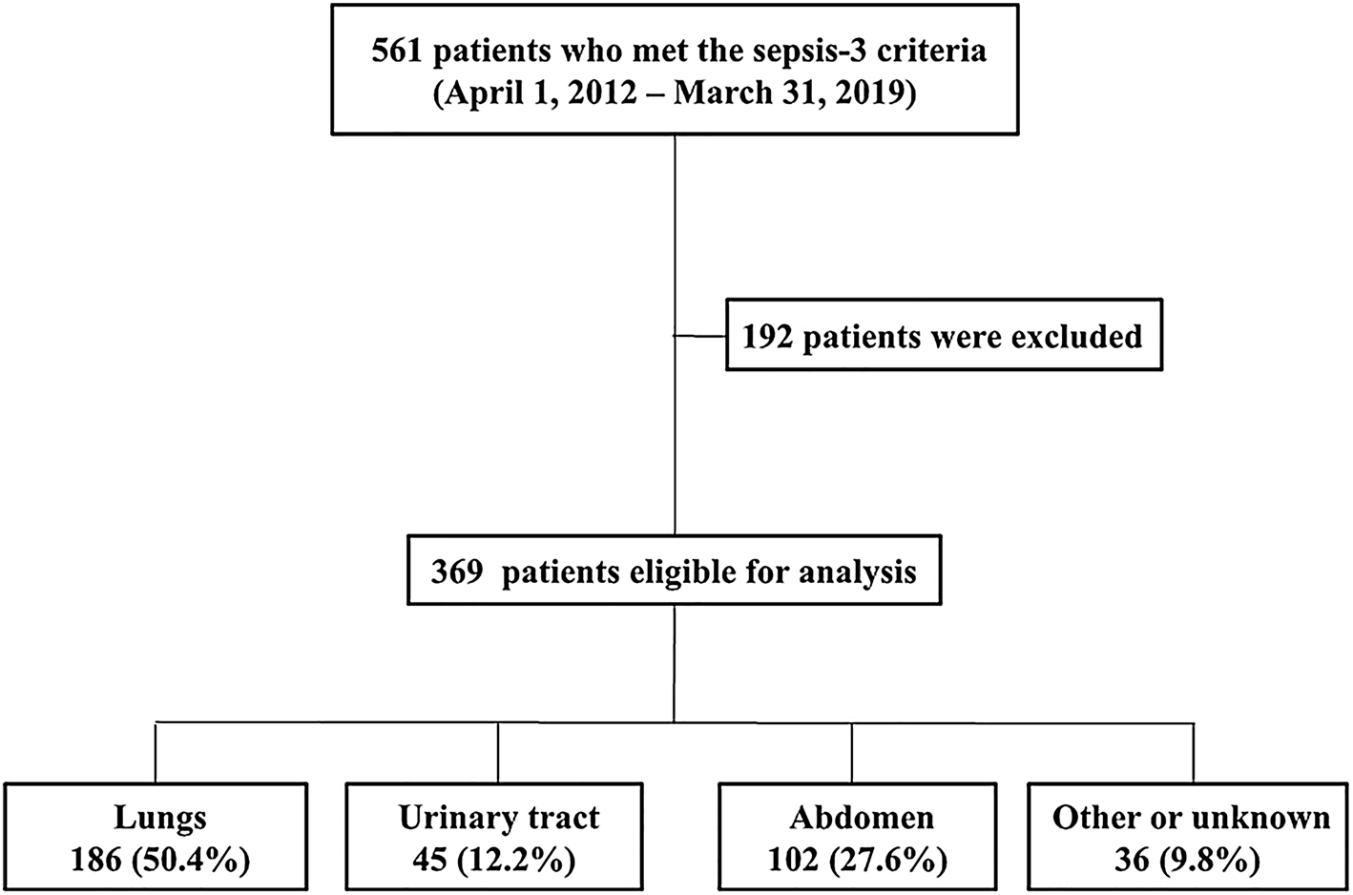
Flow diagram of patient selection.

Figure 1 shows the patient selection flowchart. A total of 369 patients with sepsis were enrolled during the study period. Supplementary Table S1 on line summarizes the baseline characteristics and outcomes according to the site of infection. There were statistically significant differences among the four groups in terms of age and sex. There were no significant differences in the initial lactate concentration and LC among the four groups. Although the difference was not statistically significant, in-hospital mortality tended to be higher in the pneumonia group compared with the urinary tract, abdomen, and other (soft tissue, central nervous system, and undifferentiated sites of infection) groups (24.2% vs. 11.1% vs. 17.6% vs. 16.7%, *p* = 0.182).

Table 1 provides a comparison of the characteristics and the results of the univariable analysis for the outcomes between the pneumonia and non-pneumonia groups. Compared with the non-pneumonia group, the pneumonia group tended to have a higher in-hospital mortality rate (24.2% vs. 15.8%, *p* = 0.051) with significantly fewer VFDs (median [interquartile range] = 20 days^1–25^ vs. 24 days^13–28^, *p* = 0.002). The *p* value for both the interaction term of pneumonia and LC on in-hospital mortality was 0.002, which indicated that pneumonia had a significant impact on the influence of LC on outcomes. All *p* values for the interaction term of site of infection category (pneumonia or non-pneumonia group) and LC were < 0.1, which indicated significant between-group differences in the impact of LC on outcomes.

**Table 1:** Comparison of characteristics and outcomes between the groups with pneumonia and with other infections.

	Pneumonia group (n = 186)	Non-pneumonia group (n = 183)	*p* value
**Characteristics**			
Age (years)	77.0 [67.8–84.0]	75.0 [66.0–84.0]	0.282
Male sex	132 (71.0%)	103 (56.3%)	0.004
SOFA scores	8.0 [6.0–10.3]	8.0 [5.0–10.0]	0.168
Initial lactate (mmol/L)	3.4 [2.1–5.6]	4.0 [2.5–6.9]	0.078
LC (%)	51.0 [17.9–68.8]	51.3 [24.1–69.0]	0.621
**Outcome**			
In-hospital mortality	45 (24.2%)	29 (15.8%)	0.051
VFD (days)	20 [1–25]	24 [13–28]	0.002

Values are expressed as: number (%) or median [interquartile range].

SD, standard deviation; IQR, interquartile range; SOFA, Sequential Organ Failure Assessment; LC, lactate clearance; VFD, ventilator-free days.

Supplementary Table S2 on line summarizes the baseline characteristics between survivors and non-survivors in the pneumonia and non-pneumonia groups. In the pneumonia group, there was no significant difference in LC between survivors and non-survivors (48.4 [17.6–70.8] vs. 52.0 [25.0–65.0], *p* = 0.554). In the non-pneumonia group, survivors had higher LC than non-survivors (54.0 [33.8–71.8] vs. 3.33 [− 34.6 to 87.1], *p* = 0.001).

Table 2 and Supplementary Tables S3 and S4 online provide the multivariate analysis results regarding the impact of LC on outcomes in the overall population and both groups. While we found a significant association after adjusting for age and SOFA scores between a lower LC and higher in-hospital mortality and less VFD in the overall population and non-pneumonia group, we did not observe a significant impact of LC on either outcome in the pneumonia group.

**Table 2:** Impact of lactate clearance on outcomes according to multivariate regression analysis.

	Pneumonia group, n = 186			Non-pneumonia group, n = 183		
	AOR [95% CI]	AD [95% CI]	*p* value	AOR [95% CI]	AD [95% CI]	*p* value
In-hospital mortality	0.99 [0.99–1.00]	–	0.134	0.97 [0.96–0.98]	–	< 0.001
VFD (days)	–	− 0.08 [− 2.06 to 1.91]	0.854	–	− 1.23 [− 2.42 to − 0.09]	0.025

AOR, adjusted odds ratio; AD, adjusted difference; CI, confidence interval; VFD, ventilator-free days.

**Figure 2:**
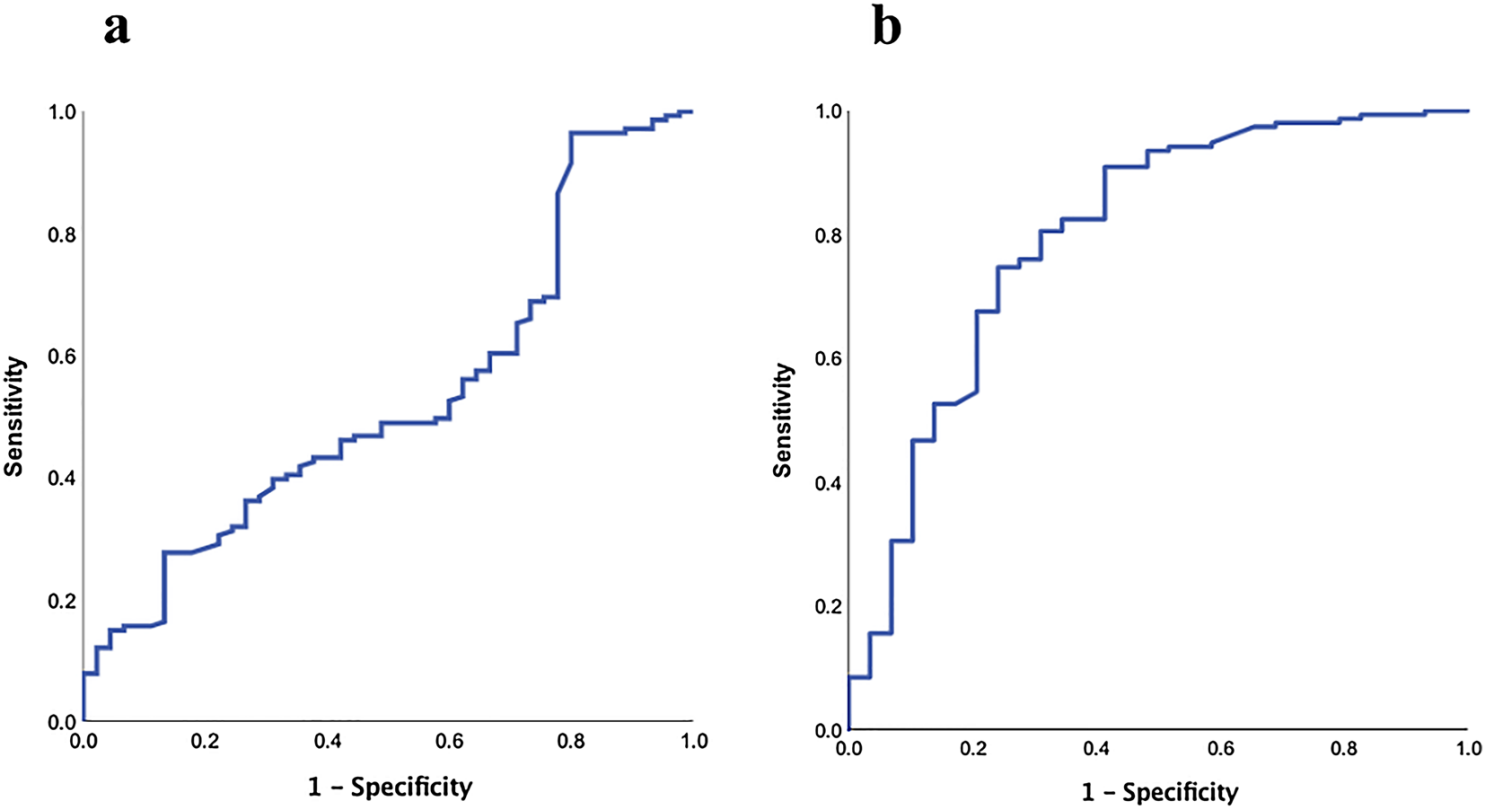
ROC of LC to predict in-hospital mortality. ROC of LC for predicting in-hospital mortality in (**a**) the pneumonia group and (**b**) non-pneumonia group. The AUC for LC was 0.529 (95% CI 0.434–0.625, *p* = 0.546) and 0.798 (95% CI 0.799–0.897, *p* < 0.001), respectively.

**Figure 3:**
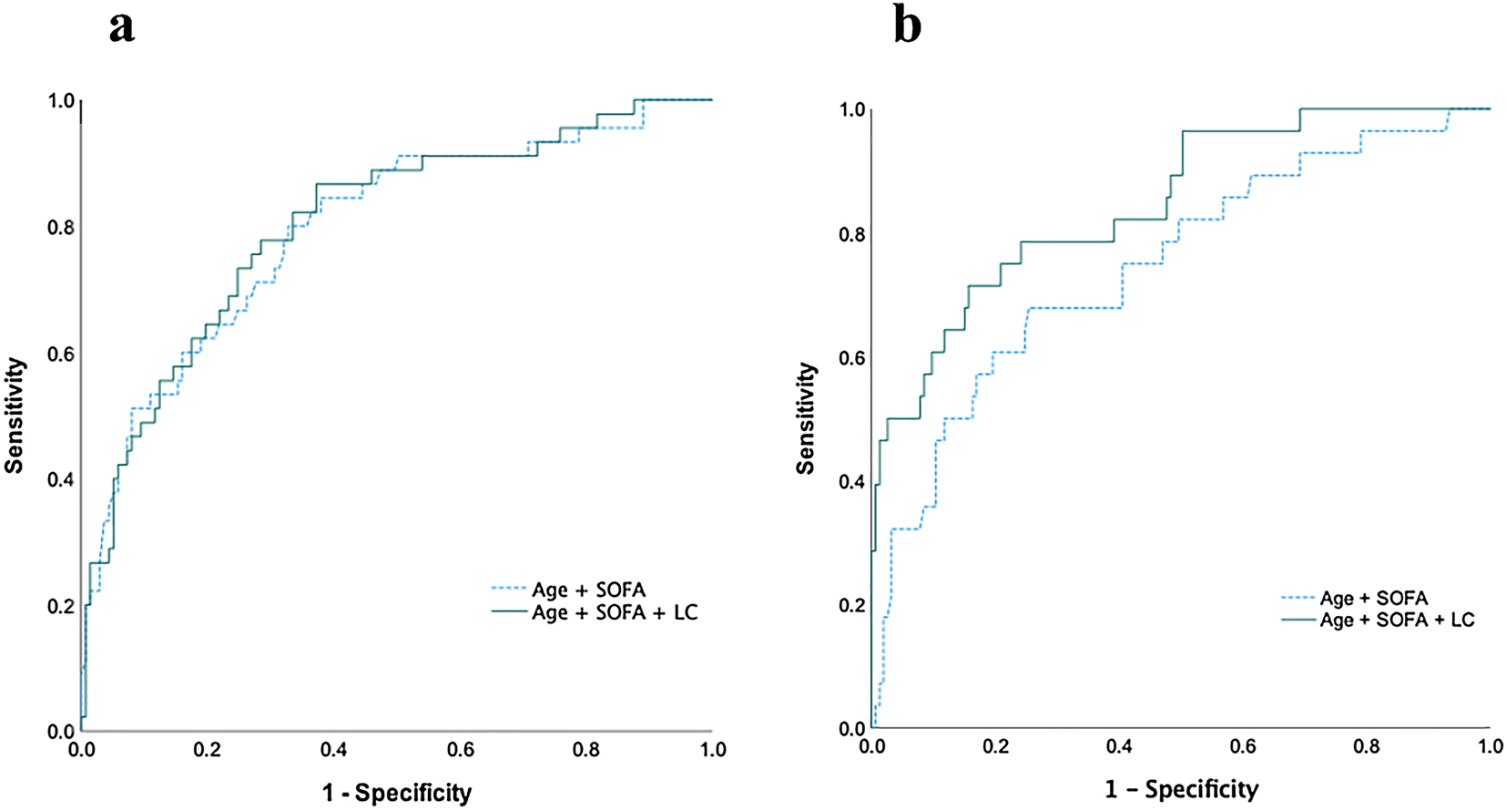
Comparison between two different models of ROC to predict in-hospital mortality. Comparison between ROC of age and SOFA scores (blue dashed line) and that of age, SOFA scores, and LC (green solid line) for in-hospital mortality in the pneumonia and non-pneumonia groups. (**a**) In the pneumonia group, the AUC for age and SOFA score was 0.796 (95% CI 0.718–0.874, *p* < 0.001), while the AUC for age, SOFA scores, and LC was 0.803 (95% CI 0.727–0.879, *p* < 0.001). (**b**) In the non-pneumonia group, the AUC for age and SOFA score was 0.746 (95% CI 0.642–0.0.850, *p* < 0.001), while the AUC for age, SOFA scores, and LC was 0.848 (95% CI 0.768–0.927, *p* < 0.001).

The ROC curve for the prediction of in-hospital mortality by LC is shown in Fig. 2. The AUC in the pneumonia group was lower than that in the non-pneumonia group (0.529 vs. 0.798) (Fig. 2a,b). Figure 3 shows the comparison of the AUCs for in-hospital mortality in each group. Although there was almost no difference between the AUC for age and SOFA scores and that for age, SOFA scores, and LC (0.796 vs. 0.803) in the pneumonia group (Fig. 3a), the AUC for age, SOFA scores, and LC was higher than that for age and SOFA scores (0.848 vs. 0.746) in the non-pneumonia group (Fig. 3b).

## Discussion

In this retrospective observational study, after adjusting for potential confounders, we observed a lower impact of LC on in-hospital mortality and VFD in the pneumonia group than in the non-pneumonia group. Notably, in the pneumonia group, adding LC to predictor variables did not improve the prognostic value for in-hospital mortality. To the best of our knowledge, this is the first study to evaluate the impact of LC on patient outcomes based on the site of infection.

Numerous studies have reported that elevated lactate levels are a strong predictor of high mortality and morbidity rates in critically ill patients^13–15^. Since the review of the Surviving Sepsis Campaign Guideline in 2016, the importance of monitoring lactate concentration to normalize lactate levels has been emphasized^1,16–18^. As lactate concentration represents the balance between production and elimination, serial lactate measurements can be more informative than a single measurement. Additionally, some studies have emphasized the utility of LC^3–5,19,20^. It has been reported that a higher LC is associated with a lower mortality and morbidity rate in patients with sepsis or septic shock^4^. Another observational cohort study of lactate-guided resuscitation in patients with septic shock in the ED showed improved clinical outcomes^7^. Furthermore, randomized controlled trials have reported the benefit of lactate-guided management in patients with sepsis^8,21^ and have suggested that a therapeutic strategy, including fluid infusion based on the results of lactate monitoring, is important^8^. However, the best time to measure lactate concentration remains controversial^22^. We measured lactate concentration at 24 h after admission, whereas some studies measured lactate concentration at 6 or 12 h after admission. The median values of LC at 24 h obtained in our study were similar to those obtained at 24 h in other studies^5^; however, the numerical values of LC measured at 24 h were higher than those measured at 6 or 12 h^3,4^, which could explain the higher impact of LC on outcomes. Some studies^3,4,20,32^ have reported that LC at 6 h is a significant factor associated with mortality rate and organ dysfunction in severe sepsis and septic shock; however, another study has reported that LC at 24 h is more associated with such negative outcomes than that at 6 or 12 h^5^. Thus, further large-scale studies are needed to detect significant differences in the timings of the measurement of lactate concentration.

The lower performance of LC in the pneumonia group in this study indirectly suggests that a judicious fluid therapy might improve outcomes in septic patients with pneumonia. Although early effective fluid resuscitation plays a decisive role in reducing lactate concentration by improving hypoperfusion in patients with sepsis^1^, fluid overload can cause pulmonary edema and respiratory failure^23^. Additionally, a conservative fluid strategy in treating acute lung injury (ALI) is associated with improved lung function and a shortened duration of mechanical ventilation and intensive care^10^. A previous study demonstrated that severe pneumonia is strongly associated with ALI^24^; therefore, fluid overload resulting from liberal fluid management to decrease lactate concentration in patients with pneumonia may be harmful.

One potential explanation for the results obtained in our study could be the difference in lactate metabolism between the lungs and other organs. An injured lung, which is often observed in patients with severe pneumonia related to ALI, can produce a larger amount of lactate than other organs^25,26^, possibly due to the large number of vascular endothelial surfaces in the lung^27^. Inflammatory cytokines directly affect pulmonary cells and glucose metabolism in cells infiltrating the lung tissue, which may result in a larger amount of lactate being produced^28^. Furthermore, neutrophils that accumulate in the pulmonary circulation can release lactate in the injured lung^29^. Although some theories have been discussed, most of the mechanisms of lactate release in the lungs remain unclear. The origins of ALI and acute respiratory distress syndrome (ARDS) may be pulmonary or extrapulmonary in nature, or both. Moreover, there are several differences between ALI and ARDS regarding epidemiology, respiratory mechanics, and long-term recovery^30^. Although we did not evaluate these factors separately in our study, whether the origin of respiratory failure was pulmonary or extrapulmonary in nature could affect the impact of LC on patient outcome.

Another possible explanation for the differences between the pneumonia and non-pneumonia groups could be the alveolar epithelial type II cells. Lactate accumulation and release could occur due to reduced capacity for lactate uptake and consumption by alveolar epithelial type II cells in the hypoxic condition of pneumonia^31^. Further basic research is warranted to elucidate these underlying mechanisms.

In this study, although the multivariate analysis results showed that LC was an independent predictor of higher mortality and shorter VFDs in the non-pneumonia group, the AORs in the non-pneumonia group were small. Although previous studies^4,32^ have reported similar results, the relatively small sample size of this study might explain these results.

In this study, we have focused on the difference between the pneumonia and non-pneumonia groups. Although the clinical applicability of the results is uncertain, clinicians can obtain significant insights regarding the utility of LC according to infection sites in patients with sepsis.

Our findings could potentially influence treatment strategies for patients with sepsis or septic shock. Although the implementation of early and quantitative resuscitation was associated with improvements in the outcomes of sepsis or septic shock, there is a potential risk in employing a fluid resuscitation strategy based on LC, especially in patients with pneumonia. It has also been reported that patients in a lactate-guided management group tended to receive a larger amount of fluid than those in a control group^8,21^.Specifically, the risk of using fluid resuscitation to decrease lactate concentration may outweigh its benefit, and the limited prediction of LC on the outcomes of patients with severe pneumonia may mislead clinicians. Further large-scale studies are needed to develop a fluid resuscitation strategy for patients with sepsis according to the sites of infection.

This study had several limitations that should be acknowledged. First, this was a retrospective study with a limited sample size, which limited the generalizability of our findings. Second, data on clinical conditions, such as renal failure and hepatic dysfunction, could not be collected, which may have affected the production and elimination of lactate. Furthermore, we did not calculate fluid balance (i.e., fluid intake and urine output). Third, we did not evaluate therapeutic interventions, including surgery, time to antimicrobial therapy, and use of vasopressors, which may have affected the outcomes. Additional studies using larger and more comprehensive datasets are necessary. Fourth, besides sepsis severity, pneumonia itself affects respiratory function, which may explain differences in VFD. Fifth, although the surviving sepsis campaign bundle was used in this study, the decisions for the response to treatment were made by clinicians. Sixth, we did not obtain the lactate concentration at times earlier than 24 h after admission (e.g., 6 or 12 h), which may have affected LC values and its impact on observed outcomes. Finally, we did not have the correct number of patients who were excluded for each criterion because we included target patients after excluding patients who did not meet the inclusion criteria. Despite these limitations, this was the first study to evaluate the difference in the impact of LC on mortality in patients with sepsis according to the organ of infection. Further studies are warranted to validate our findings and improve therapeutic interventions for patients with sepsis.

In conclusion, among patients with sepsis, the ability of LC to predict outcomes in patients with pneumonia was weaker than that in patients with other infections. Given the difference in utility of LC according to infection sites, this study suggests that optimal treatment strategies might improve outcomes, and further research to validate our findings is needed.

## Supplementary Information

Supplementary Information 1.

Supplementary Information 2.

Supplementary Information 3.

Supplementary Information 4.

## Data Availability

The datasets used and/or analyzed during the current study are not publicly available because of privacy issues but are available from the corresponding author upon reasonable request.

## References

[1] Rhodes A (2017). Surviving sepsis campaign: international guidelines for management of sepsis and septic shock: 2016. Intensive Care Med..

[2] Vincent JL, De Backer D (2013). Circulatory shock. N. Engl. J. Med..

[3] Nguyen HB (2010). Early lactate clearance is associated with biomarkers of inflammation, coagulation, apoptosis, organ dysfunction and mortality in severe sepsis and septic shock. J. Inflamm. (Lond.).

[4] Nguyen HB (2004). Early lactate clearance is associated with improved outcome in severe sepsis and septic shock. Crit. Care Med..

[5] Marty P (2013). Lactate clearance for death prediction in severe sepsis or septic shock patients during the first 24 hours in intensive care unit: an observational study. Ann. Intensive Care.

[6] Wang H (2009). Relationship between blood lactic level, lactic clearance, duration of lacticemia and prognosis of critically ill patients in intensive care unit. Zhongguo Wei Zhong Bing Ji Jiu Yi Xue.

[7] Dettmer M, Holthaus CV, Fuller BM (2015). The impact of serial lactate monitoring on emergency department resuscitation interventions and clinical outcomes in severe sepsis and septic shock: an observational cohort study. Shock.

[8] Jansen TC (2010). Early lactate-guided therapy in intensive care unit patients: a multicenter, open-label, randomized control trial. Am. J. Respir. Crit. Care Med..

[9] Casserly B (2015). Lactate measurements in sepsis-induced tissue hypoperfusion: results from the Surviving Sepsis Campaign database. Crit. Care Med..

[10] National Heart, Lung, and Blood Institute Acute Respiratory Distress Syndrome (ARDS) Clinical Trials Network et al. Comparison of two fluid-management strategies in acute lung injury. *N. Engl. J. Med.* **354**, 2564–2575 (2006).

[11] Singer M (2016). The third international consensus definitions for sepsis and septic shock (Sepsis-3). JAMA.

[12] Schoenfeld, D. A., Bernard, G. R. & ARDS Network. Statistical evaluation of ventilator-free days as an efficacy measure in clinical trials of treatments for acute respiratory distress syndrome. Crit. Care Med. 30, 1772–1777 (2002). 10.1097/00003246-200208000-00016 12163791

[13] Bakker J, Gris P, Coffernils M, Kahn RJ, Vincent JL (1996). Serial blood lactate levels can predict the development of multiple organ failure following septic shock. Am. J. Surg..

[14] Bakker J, Coffernils M, Leon M, Gris P, Vincent JL (1991). Blood lactate levels are superior to oxygen-derived variables in predicting outcome in human septic shock. Chest.

[15] Bernardin G, Pradier C, Tiger F, Deloffre P, Mattei M (1996). Blood pressure and arterial lactate level are early indicators of short-term survival in human septic shock. Intensive Care Med..

[16] Investigators ProCESS (2014). A randomized trial of protocol-based care for early septic shock. N. Engl. J. Med..

[17] ARISE Investigators et al. Goal-directed resuscitation for patients with early septic shock. N. Engl. J. Med. 371, 1496–1506 (2014). 10.1056/NEJMoa1404380 25272316

[18] Mouncey PR (2015). Trial of early, goal-directed resuscitation for septic shock. N. Engl. J. Med..

[19] Puskarich MA (2012). Prognostic value and agreement of achieving lactate clearance or central venous oxygen saturation goals during early sepsis resuscitation. Acad. Emerg. Med..

[20] Walker CA, Griffith DM, Gray AJ, Datta D, Hay AW (2013). Early lactate clearance in septic patients with elevated lactate levels admitted from the emergency department to intensive care: time to aim higher?. J. Crit. Care..

[21] Jones AE (2010). Lactate clearance vs central venous oxygen saturation as goals of early sepsis therapy: a randomized clinical trial. JAMA.

[22] Vincent JL (2016). The value of blood lactate kinetics in critically ill patients: a systematic review. Crit. Care..

[23] Alsou F, Khamiees M, DeGirolamo A, Amoateng-Adjepong Y, Manthous CA (2000). Negative fluid balance predicts survival in patients with septic shock: a retrospective pilot study. Chest.

[24] Rubenfeld GD (2005). Incidence and outcomes of acute lung injury. N. Engl. J. Med..

[25] Brown SD, Clark C, Gutierrez G (1996). Pulmonary lactate release in patients with sepsis and ARDS. J. Crit. Care..

[26] De Backer D, Creteur J, Zhang H, Norrenberg M, Vincent JL (1997). Lactate production by the lungs in acute lung injury. Am. J. Respir. Crit. Care Med..

[27] Kellum JA (1997). Release of lactate by the lung in acute lung injury. Chest.

[28] Iscra F, Gullo A, Biolo G (2002). Bench-to-bedside review: Lactate and the lung. Crit. Care.

[29] Kelm DJ (2015). Fluid overload in patients with severe sepsis and septic shock treated with early goal-directed therapy is associated with increased acute need for fluid-related medical interventions and hospital death. Shock.

[30] Pelosi P (2003). Pulmonary and extrapulmonary acute respiratory distress syndrome are different. Eur. Respir. J..

[31] Lottes RG (2015). Lactate as substrate for mitochondrial respiration in alveolar epithelial type II cells. Am. J. Physiol. Lung. Cell. Mol. Physiol..

[32] Ryoo SM (2018). Lactate level versus lactate clearance for predicting mortality in patients with septic shock defined by sepsis-3. Crit. Care Med..

