# Supplementary material for: The impact of lactate clearance on outcomes according to infection sites in patients with sepsis: a retrospective observational study
Source: Sci Rep. 2021 Nov 17;11:22394. doi: 10.1038/s41598-021-01856-5 (PMC8599851; doi:10.1038/s41598-021-01856-5)
Supplement: Supplementary file 1 — Supplementary Information 1. [file 41598_2021_1856_MOESM1_ESM.docx]

| **Supplementary Table 1: Baseline characteristics and outcomes in each infected organ group** | | | | | | |
| --- | --- | --- | --- | --- | --- | --- |
|  | | **Lungs (n=186)** | **Urinary tract (n=45)** | **Abdomen (n=102)** | **Other or unknown (n=36)** | ***p* value** |
| **Characteristics** | | | | | | |
|  | Age (y) | 77.0 [67.8–84.0] | 80.0 [73.5–88.0] | 74.0 [65.8–83.3] | 68.5 [61.0–80.5] | <0.001 |
|  | Male sex | 132 (71.0%) | 18 (40.0%) | 63 (61.8%) | 22 (61.1%) | 0.001 |
|  | SOFA scores | 8.0 [6.0–10.3] | 9.0 [6.5–11.0] | 7.0 [4.0–10.0] | 7.0 [5.0–12.0] | 0.080 |
|  | Initial lactate (mmol/L) | 3.4 [2.1–5.6] | 3.9 [2.7–6.6] | 3.9 [2.5–7.3] | 4.1 [2.6–6.3] | 0.357 |
|  | LC (%) | 51.0 [18.0–68.5] | 55.0 [28.0–68.0] | 51.7 [25.6–68.0] | 40.0 [15.5–73.2] | 0.738 |
| **Outcomes** | | | | | | |
|  | In-hospital mortality | 45 (24.2%) | 5 (11.1%) | 18 (17.6%) | 6 (16.7%) | 0.182 |
|  | VFD (days) | 20 [1–25] | 27 [23–28] | 22 [5–26] | 24 [8–28] | <0.001 |

Values are expressed as number (%) or median [interquartile range].

Abbreviations: IQR, interquartile range; SOFA, Sequential Organ Failure Assessment; LC, lactate clearance; VFD, ventilator-free days.
