# Supplementary material for: The impact of lactate clearance on outcomes according to infection sites in patients with sepsis: a retrospective observational study
Source: Sci Rep. 2021 Nov 17;11:22394. doi: 10.1038/s41598-021-01856-5 (PMC8599851; doi:10.1038/s41598-021-01856-5)
Supplement: Supplementary file 2 — Supplementary Information 2. [file 41598_2021_1856_MOESM2_ESM.docx]

| **Supplementary Table 2: Baseline characteristics between survivors and non-survivors** | | | | |
| --- | --- | --- | --- | --- |
| **Pneumonia group** | | | | |
|  | | **Survivors (n=141)** | **Non-survivors (n=45)** | ***p* value** |
|  | Age (y) | 75.0 [65.0–82.0] | 82.0 [74.0–88.0] | 0.002 |
|  | Male sex | 101 (71.6%) | 31 (68.9%) | 0.710 |
|  | SOFA scores | 7.0 [5.0–9.0] | 11.0 [8.0–13.0] | <0.001 |
|  | Initial lactate (mmol) | 3.3 [1.9–4.9] | 5.5 [2.8–8.2] | <0.001 |
|  | LC (%) | 48.4 [17.6–70.8] | 52.0 [25.0–65.0] | 0.554 |
| **Non-pneumonia group** | | | | |
|  | Age (y) | 75.0 [65.3–84.0] | 76.0 [67.0–82.0] | 0.752 |
|  | Male sex | 86 (55.8%) | 17 (58.6%) | 0.840 |
|  | SOFA scores | 7.0 [4.0–9.0] | 11.5 [7.8–14.0] | <0.001 |
|  | Initial lactate (mmol) | 3.9 [2.5–5.9] | 7.2 [3.5–10.1] | 0.002 |
|  | LC (%) | 54.0 [33.8–71.8] | 3.33 [-34.6–87.1] | <0.001 |
| Values are expressed as number (%) or median [interquartile range].  Abbreviations: SOFA, Sequential Organ Failure Assessment; LC, lactate clearance | | | | |
