# Supplementary material for: The impact of lactate clearance on outcomes according to infection sites in patients with sepsis: a retrospective observational study
Source: Sci Rep. 2021 Nov 17;11:22394. doi: 10.1038/s41598-021-01856-5 (PMC8599851; doi:10.1038/s41598-021-01856-5)
Supplement: Supplementary file 3 — Supplementary Information 3. [file 41598_2021_1856_MOESM3_ESM.docx]

| **Supplementary Table 3: Multivariate analysis of factors influencing outcomes and the comparison of explanatory variables in overall population** | | | | | |
| --- | --- | --- | --- | --- | --- |
|  | | | **Adjusted odds ratio**  **[95% CI]** | **Adjusted difference *p* value [95% CI]** | ***p* value** |
| **Overall population** | | | | | |
|  | In-hospital mortality | | | | |
|  |  | age | 1.05 [1.02 – 1.08] | - | <0.001 |
|  |  | SOFA scores | 1.34 [1.22 – 1.47] | - | <0.001 |
|  |  | lactate clearance | 0.99 [0.98 – 0.99] | - | <0.001 |
|  | VFD | | | | |
|  |  | age | - | -1.4 [-1.6 – -0.9] | 0.028 |
|  |  | SOFA scores | - | -1.2 [-1.5 – -0.6 ] | 0.038 |
|  |  | lactate clearance | - | -0.98 [-2.12 – - 0.09] | 0.013 |
| CI, confidence interval; SOFA, sequential organ failure assessment; VFD, ventilator-free days | | | | | |
