# Supplementary material for: The impact of lactate clearance on outcomes according to infection sites in patients with sepsis: a retrospective observational study
Source: Sci Rep. 2021 Nov 17;11:22394. doi: 10.1038/s41598-021-01856-5 (PMC8599851; doi:10.1038/s41598-021-01856-5)
Supplement: Supplementary file 4 — Supplementary Information 4. [file 41598_2021_1856_MOESM4_ESM.docx]

| **Supplementary Table 4: Multivariate analysis of factors influencing outcomes and the comparison of explanatory variables** | | | | | |
| --- | --- | --- | --- | --- | --- |
|  | | | **Adjusted odds ratio**  **[95% CI]** | **Adjusted difference *p* value [95% CI]** | ***p* value** |
| **Pneumonia group** | | | | | |
|  | In-hospital mortality | | | | |
|  |  | age | 1.05 [1.02 – 1.09] | - | 0.006 |
|  |  | SOFA scores | 1.40 [1.23 – 1.59] | - | <0.001 |
|  |  | lactate clearance | 0.99 [0.99 – 1.00] | - | 0.134 |
|  | VFD | | | | |
|  |  | age | - | -1.7 [-2.5 – -0.9 ] | 0.023 |
|  |  | SOFA scores | - | -1.3 [-1.4 – -0.8] | 0.041 |
|  |  | lactate clearance | - | -0.08 [-2.06 – 1.91] | 0.854 |
| **Non-pneumonia group** | | | | | |
|  | In-hospital mortality | | | | |
|  |  | age | 1.05 [1.00 – 1.10] | - | 0.039 |
|  |  | SOFA scores | 1.29 [1.12 – 1.50] | - | 0.001 |
|  |  | lactate clearance | 0.97 [0.96 – 0.98] | - | <0.001 |
|  | VFD | | | | |
|  |  | age | - | -1.2 [-1.4 – -0.7] | 0.031 |
|  |  | SOFA scores | - | -0.9 [-1.2 – -0.6 ] | 0.041 |
|  |  | lactate clearance | - | -1.23 [-2.42 – - 0.09] | 0.025 |
| CI, confidence interval; SOFA, sequential organ failure assessment  AOR, adjusted odds ratio; AD, adjusted difference; VFD, ventilator-free days | | | | | |
